# Causes of death and associated risk factors among climacteric women from Southern Brazil: a population based-study

**DOI:** 10.1186/1471-2458-14-194

**Published:** 2014-02-21

**Authors:** Verônica Colpani, Karen Oppermann, Poli Mara Spritzer

**Affiliations:** 1 Gynecological Endocrinology Unit, Division of Endocrinology, Hospital de Clínicas de Porto Alegre, Porto Alegre, RS, Brazil; 2 Medical School of Universidade de Passo Fundo and São Vicente de Paulo Hospital, Rua Teixeira Soares 885/704, CEP 99010-081 Passo Fundo, RS, Brazil; 3 Laboratory of Molecular Endocrinology, Department of Physiology, Universidade Federal do Rio Grande do Sul, Porto Alegre, RS, Brazil

**Keywords:** Menopause, Mortality, Risk factors

## Abstract

**Background:**

Aging and menopause are particular cardiovascular risk factors for women, due to estrogen deprivation at the time of menopause. Studies show that diabetes mellitus (DM), smoking, hypertension, high body mass index (BMI), and serum lipids are associated with increased risk of cardiovascular disease (CVD), the main cause of female mortality in Brazil. The aim of this study was to assess the mortality rate, causes of death and associated risk factors in a cohort of women from Brazil.

**Methods:**

A longitudinal population-based study of menopausal status is currently underway in a city in South Brazil. In 2010, a third follow-up of this population was performed to assess cardiovascular risk and mortality rate between 1995 and 2011. For this analysis, 358 participants were studied. At baseline, participants had completed a standardized questionnaire including demographic, lifestyle, medical and reproductive characteristics. In addition to the contacts with relatives, mortality data were obtained through review of medical records in all city hospitals and the Center for Health Information (NIS/RS-SES). Multivariate-adjusted hazard risk (HR) and 95% confidence intervals (CI95%) were estimated using Cox proportional hazards regression. Survival curves were estimated using the Kaplan-Meier curve.

**Results:**

There were 17 (4.7%) deaths from all causes during the study period. Seven (41.2%) deaths were caused by CVD, including four cases of stroke and three cases of myocardial infarction. Six (35.3%) deaths were due to cancer, and four (23.5%) were due to other reasons. In the age and smoking-adjusted multivariate models, diabetes (HR 6.645, 95% CI: 1.938–22.79, p = 0.003), alcohol intake (HR 1.228, 95% CI: 1.014-1.487, p = 0.035) and postmenopausal status (HR = 6.216, 95% CI: 0.963–40.143, p = 0.055) were associated with all-cause mortality. A significant association was found between abdominal obesity (WHR ≥ 0.85) and mortality even after the adjustment for BMI (HR = 9.229, 95% IC: 2.083–41.504, p = 0.003).

**Conclusion:**

CVD was an important cause of mortality in this cohort and DM and/or central adiposity were associated with all-cause mortality. Lifestyle and dietary factors seem to be related to risk of mortality in middle-aged women.

## Background

Life expectancy is increasing in the world as well as in Brazil, where recent data from the National Geography and Statistics Institute [1] show that females and males are now expected to reach 77.3 and 69.7 years of age respectively, as compared to 72.9 and 65.1 years only a decade ago. This population aging process will have an impact on health and social policies. However, only a few studies are available about middle-aged female mortality, especially in Brazil [2,3], where a vast territory and socioeconomic diversity contribute to a scenario of public health inequity. Cultural and economic differences may influence diet, health, and behavioral factors and consequently mortality rates. Thus, knowledge of the pattern of mortality risk is useful to support actions of prevention and control.

Aging and menopause may be considered as particular cardiovascular risk factors for women, due to estrogen deprivation at the time of menopause [4]. Also, studies have consistently shown that diabetes mellitus (DM) [5,6], smoking [7,8], hypertension [9,10], high body mass index (BMI) [11-13] and serum lipids [14] are associated with increased CVD risk. Therefore, even though CVD mortality has decreased in recent decades [15,16] following improvements in prevention, diagnosis, and timing of treatment [17], along with gradual improvement in economic conditions, more widespread access to drugs [18], heath surveillance, and policies of health promotion [15], CVD remains a major cause of death [15], and the main cause of female mortality in Brazil [15,17,19].

Based on these data, and on the scarcity of literature about this subject, the present study aims to assess mortality, CVD risk factors and causes of death in a cohort of pre-, peri- and postmenopausal women in the South of Brazil.

## Methods

### Study population

Participants were selected from the population-based cohort of the longitudinal menopause study that has been underway in the city of Passo Fundo, South Brazil, since 1995 [20,21]. As part of this project, an initial cross-sectional study was performed between 1995 and 1997 to investigate ovarian volume in pre- and perimenopausal women. Sampling was carried out in two stages. First, 154 census sections (geographical subdivisions of the city defined by the Brazilian Institute of Geography and Statistics) were randomly selected. One block in each census section was picked by lot; two women were interviewed in each block after the randomization method described previously [20,21]. A representative sample of 298 women aged 35 to 55 years who had menstruated at least once in the past 12 months was randomly identified.

**Figure 1:**
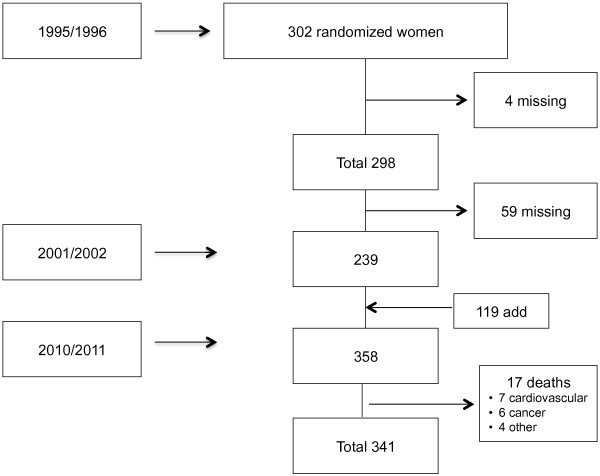
Flowchart of the process of study selection and lost to follow-up in the three phases of the study.

In the second field visit, conducted between 2001 and 2002, 239 women from the baseline cohort were located and interviewed. In view of potential losses to follow-up and of the increasing city population, 119 additional women aged 35 to 62 years were sampled to guarantee enough statistical power for the analysis. The final sample was 358 women. The samples were selected at random based on the first census sections [22] (Figure 1).

### Mortality, causes of death, and CVD risk factors

In 2010, a third follow-up was initiated in order to assess cardiovascular risk and mortality rate. All 358 participants or their relatives were reached, and information regarding participant deaths was obtained for the period ending in November 2011. In addition to the interviews, the medical records of city hospitals and the Center for Health Information (NIS/RS-SES) were reviewed. All deaths between 1995 and 2011 were included in this analysis.

Medical records were reviewed to collect information on age at death, date, and cause of death. The causes of death were coded using the International Classification of Diseases, 10th revision [23]. Deaths were analyzed for all-cause and cardiovascular causes (ICD-10: I00-I99), neoplasms (ICD-10: C00-C97), and respiratory causes (ICD-10: J00-J99).

Ethics approval for the study was granted by the Research Ethics Committees at the University of Passo Fundo and the São Vicente de Paulo Hospital. All study participants signed an informed consent.

### Social characteristics

The participants were interviewed using a pretested standardized questionnaire covering demographic characteristics (age and self-reported race) and education (years of successful formal education, described as years at school). Skin color was classified according to self-report [21,22]. Women were classified in terms of alcohol consumption as nondrinkers, social drinkers (1 to 15 g alcohol/day), or abusers (at least 15 g alcohol/day) [24]. Smoking status was categorized as current, ex-smoker, or nonsmoker [22]. Physical activity was investigated through a previously tested standardized questionnaire [25]; for each type of physical activity, the metabolic equivalent [26] and overall calorie expenditure were calculated. Women who expended at least 1000 kcal/week (approximately 3.5 hours per week walking, climbing stairs, swimming, playing sports, doing yard work, and so forth) were considered to be physically active, whereas the others were classified as sedentary [22].

Women were classified according to their baseline menopausal status: premenopause was defined as no change in menstrual frequency or flow; perimenopause was defined as changes in menstrual frequency or flow in the 12 months before the study; and postmenopause was defined as 12 months or more of amenorrhea, including as a result of medical interventions such as bilateral oophorectomy [21,22]. A "hysterectomy" category was created for women who had undergone hysterectomy and whose menopausal status could not be classified [27].

The use of hormone therapy (HT), estrogen, estrogen plus progestogen or tibolone was verified by asking the women to show the medication box or the physician’s prescription [21,22].

### Anthropometric measurements

Body weight and height was assessed at the beginning of the study in 1995. Weight (kg) was measured to the nearest 100 g using a Filizola® scale, Model 31 (Ind Filizola-SA, São Paulo, Brazil), and height (cm) was measured to the nearest 0.1 cm with a wall-mounted fixed stadiometer. Special attention was taken to ensure that the participants were positioned with the Frankfort plane [28] horizontal and that they were barefoot. These were used to calculate BMI, dividing weight in kilograms by height squared (m^2^), and categorized as <25.0, 25.0–29.9, and ≥30.0 kg/m^2^[29].

Other anthropometric measurements were made in duplicate between 2001 and 2003, including waist circumference (WC) (measured at the midpoint between the lower rib margin and the iliac crest, perpendicularly to the long axis of the body, with the participant standing balanced on both feet, spread approximately 20 cm apart, with arms hanging freely), hip circumference (widest circumference over the buttocks), and waist to hip ratio (WHR) (waist circumference divided by hip circumference) [21,22,28]. All procedures followed standardized recommendations [30] and the equipment was periodically calibrated.

### Clinical variables

Previous diagnosis of DM was verified based on physician report or current use of anti-diabetic medication. Self-report of hypercholesterolemia or use of anti-cholesterol medication was used to define dyslipidemia.

Blood pressure was measured after a 10-minute rest. The same calibrated mercury manometer attached to a 12.5 × 23 cm inflatable cuff was used in all participants, and the fifth Korotkoff sound was adopted to determine diastolic pressure. Hypertension was defined as systolic blood pressure ≥140 and/or diastolic blood pressure ≥90 mmHg or current use of antihypertensive medication [31,32].

All participants were submitted, in 2003, to blood sampling between 8 and 10 a.m. after an overnight fast of 10 to 12 hours. Total cholesterol, high-density cholesterol (HDL-c), triglycerides, and glucose levels were determined by a colorimetric-enzymatic method (Architect C800, ABBOTT Systems). Low density lipoprotein cholesterol (LDL-c) was determined indirectly using the following formula: LDL-c = total cholesterol - (HDL-c + triglycerides/5) [33,34].

### Statistical analyses

Continuous variables are reported as means ± SDs. Categorical variables are reported as frequencies (%). Differences in baseline clinical characteristics between groups were analyzed by the Student *t* test (for continuous variables with normal distribution), Mann-Whitney’s U test (for continuous variables with skewed distribution), or χ^2^ test (for categorical variables). Survival time for each participant was defined as the time between the date of study entry and the occurrence of death. Univariate predictors of mortality during follow-up were analyzed by Cox regression models and calculation of hazard rate ratios with 95% CI. For the estimation of hazard ratios, a Cox regression model was fit including relevant independent variables to estimate the associations between baseline characteristics and mortality. Multivariate analysis included only variables with a p-value of 0.05 or lower on univariate analysis. Adjusted hazard ratios (HR) and 95% confidence intervals (CI95%) for mortality were estimated. We calculated adjusted hazard ratios after multivariate correction for age and smoking. The final model included all variables with P < 0.05 or according to clinical plausibility. Collinearity for the model variables was evaluated using variance inflation factors and tolerances. No collinearity was found. Survival curves were estimated using the Kaplan-Meyer method. Log-rank p-values were calculated to test for significant differences between mortality and DM. All statistics analyses were performed using SPSS 20.0 software. *P* < 0.05 was considered statistically significant for all analyses.

## Results

### Baseline characteristics

A total of 358 women were studied during a mean follow-up of 13.4 ± 3.3 years. Table 1 shows the distribution of baseline characteristics in survivors and non-survivors. The mean age of the overall sample was 44.29 ± 6.0 years. Most participants were white (86.3%), with low levels of schooling (8.4 ± 4.7 years). Considering menopause status, 162 (47.1%) were premenopausal, 134 (39%) were perimenopausal, 37 (10.3%) were postmenopausal, and 11 (3.2%) had undergone a hysterectomy. Of the 358 individuals included in the study, 177 (49.4%) reported having hypertension; 56 (15.6%) used HT, and 14 (3.9%) were diabetic. Smoking, a well-known risk factor for CVD, was found in 96 (26.8%) of the overall sample, and in 8 (47.1%) deceased patients. Survivors were significantly younger and almost half were premenopausal. They also had higher education level and fewer cases of previous DM.

**Table 1:** Demographic characteristics of participants at baseline

**Characteristic**	**Overall group**	**Survivors**	**Non-survivors**	**p**^**a**^
	**(n = 358)**	**(n = 341)**	**(n = 17)**	
Age (years)	44.29 ± 6.01	44.13 ± 6.01	48.00 ± 4.48	0.009
White skin color (yes)	86.3	87.0	76.5	0.263
Educational level (years)	8.44 ± 4.77	8.55 ± 4.73	6.29 ± 5.21	0.062
0-4	24.6	22.9	52.9	0.046
5-8	28.5	29.1	17.6	
9-11	21.5	21.8	17.6	
≥12	25.4	26.2	11.8	
Menopausal status				
Premenopause	47.1	48.2	23.5	0.040
Perimenopause	39.0	38.8	41.2	
Postmenopause	10.3	9.8	29.4	
Hysterectomy	3.2	3.1	5.9	
Smoker (yes)	26.8	26.0	47.1	0.055
Alcohol intake (g)	0.18 (0-1.78)	0.21 (0-1.82)	0.08 (0-0.57)	0.213
Nondrinkers	29.3	28.5	41.2	0.480
Social drinkers	66.5	67.4	52.9	
Abusers	4.2	4.1	5.9	
Hypertension (yes)	49.4	49.3	52.9	0.767
Diabetes (yes)	3.9	2.9	23.5	<0.001
Dyslipidemia (yes)	6.7	6.5	11.8	0.395
Hormonal therapy	15.6	15.0	29.4	0.109

^a^Continuous variables were compared using the Student’s t-test and expressed as means ± standard deviation or median (IQ). Categorical variables were compared using Pearson’s chi-square test and expresses as percentage.

Table 2 describes the metabolic profile of participants. In general, they had low level of physical activity during their leisure time and showed baseline overweight and central adiposity. A relatively healthy lipid profile was observed, with normal or borderline values. WHR and HDL-c were significantly different in survivors and non-survivors.

**Table 2:** Anthropometric and metabolic characteristics of participants at baseline

**Characteristic**	**Overall group**	**Survivors**	**Non-survivors**	**p**^**a**^
	**(n = 358)**	**(n = 341)**	**(n = 17)**	
LPA (MET)^c^	5.68 (0-13.85)	5.60 (0-13.40)	7.41 (1.6-17.92)	0.482
≥2000 cal/sem	7.5	7.4	11.8	0.051
1000-1999 cal/sem	16.2	15.9	23.5	
<1000 cal/sem	76.3	76.8	64.7	
BMI (kg/m2)^b^	27.35 ± 5.46	27.39 ± 5.55	26.60 ± 3.11	0.343
≤ 24.9	37.7	38.4	23.5	0.968
25-29.9	35.8	34.3	64.7	
≥ 30.0	26.5	27.3	11.8	
WHR (cm)^c^	0.83 ± 0.075	0.83 ± 0.072	0.90 ± 0.09	<0.001
≥ 0.85	43.0	41.3	76.5	0.004
WC (cm)^c^	85.28 ± 12.25	85.11 ± 12.35	88.73 ± 9.66	0.235
≥ 88	38.3	37.6	47.1	0.445
TC (mg/dl)^c^	200.01 ± 41.64	200.70 ± 40.03	198.0 ± 47.68	0.794
≥ 200	48.0	47.8	50.0	0.872
HDL-c (mg/dl)^c^	52.45 ± 10.91	52.35 ± 10.54	57.68 ± 9.97	0.049
<50	40.7	41.3	25.0	0.190
LDL-c (mg/dl)^c^	122.11 ± 35.89	119.95 ± 39.34	114.50 ± 41.18	0.386
≥ 160	14.9	14.6	12.5	0.783
TG (mg/dl)^c^	114.0 (79.25-157.5)	114.0 (79.25-159.75)	116.5 (73.25-150.5)	0.886
≥ 150	27.4	27.5	23.5	0.716
GLU(mg/dL)^c^	79.0 (71.0-86.0)	79.0 (70.25-86.0)	81.0 (72.5-93.5)	0.258
≥ 126	4.5	4.1	12.5	0.114

^a^Continuous variables were compared using the Student’s t-test and Mann-Whitney’s U test and expressed as means ± standard deviation or median (IQ). Categorical variables were compared using Pearson’s chi-square test and expressed as percentage. ^b^Data from baseline. ^c^Data from 2003 interviews. BMI: body mass index; GLU: levels of plasma glucose; HDL-c: high-density lipoprotein cholesterol; LDL-c: light-density lipoprotein cholesterol; LPA: leisure physical activity in the previous year; MET: metabolic equivalent; TC: plasma total cholesterol; TG: triglycerides; WC: waist circumference; WHR: waist-to-hip ratio.

### Causes of death

The mean age at death was 57.8 ± 5.5 years. In the 13 years analyzed, 17 (4.7%), deaths from all causes were recorded. Among these, seven (41.2%) deaths were caused by CVD, including four cases of stroke and three myocardial infarctions. There were six (35.3%) deaths due to kidney (n = 1), breast (n = 1), lung (n = 2) and uterine (n = 2) cancer, and four (23.5%) deaths due to other reasons, such as polytrauma, asthma, and diabetes.

### Survival estimates

**Figure 2:**
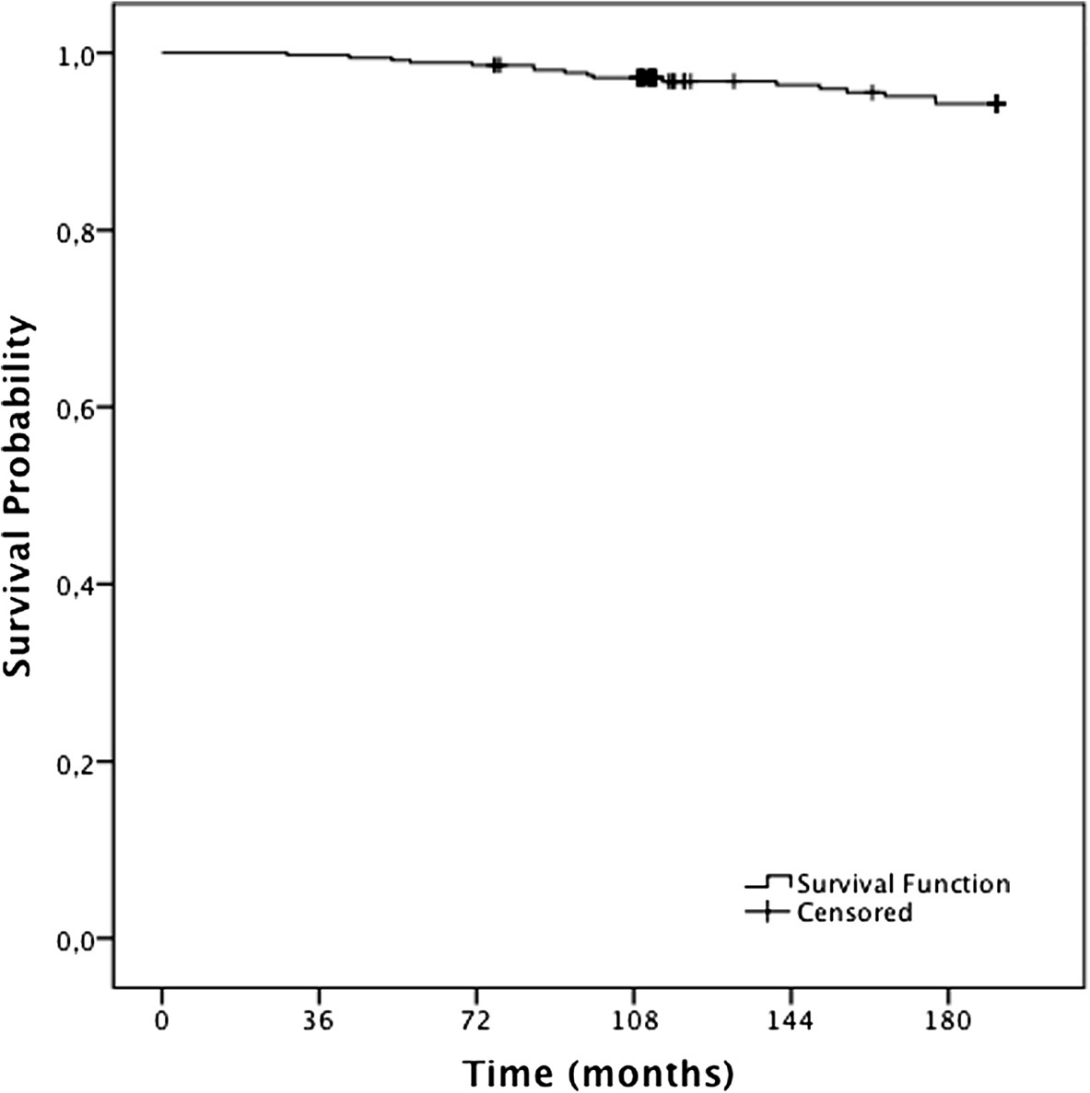
Kaplan-Meier estimates of survival among women (n = 358).

The survival for the entire cohort in 6, 12 and 15 years was 98.6%, 96.8% and 94.3%, respectively (Figure 2).

In a univariate analysis (Table 3), older age (HR = 1.141, 95% IC: 1.051-1.239, p = 0.002), menopausal status (postmenopause HR = 16.738, 95% CI: 3.644-16.895, p < 0.001), hysterectomy HR = 11.788, 95% CI: 3.644-121.382, p = 0.038), DM (HR = 10.439, 95% CI: 3.367– 32.367, p < 0.001), and WHR ≥ 0.85 (HR = 3.556, 95% CI: 1.253-10.094, p = 0.017) were associated with a major probability of death. Years at school was statistically meaningful in the univariate model (HR = 0.888, 95% CI: 0,792-0.997, p = 0.044), suggesting a protective association of higher level of education.

**Table 3:** Crude mortality hazard ratio (HR) and 95% confidence intervals (95% CI) for survival in 358 women from a South Brazilian cohort between 1995-2010

**Predicting factor**	**HR**	**95% CI**	**p**
Age (years)	1.141	1.051-1.239	0.002
Years at school^1^	0.888	0.792-0.997	0.044
0 – 4	5.240	1.131-24.275	0.034
5 – 8	1.455	0.243-8.709	0.682
9 – 11	1.869	0.312-11.184	0.493
White skin color (yes)	0.424	0.137-1.308	0.135
Menopausal status^2^			
Perimenopause	2.495	0.729	0.145
Postmenopause	16.738	3.644	<0.001
Hysterectomy	11.788	1.145	0.038
Smoker^3^ (yes)	2.465	0.951-6.389	0.063
Alcohol intake^4^			
Social Drinker	0.536	0.199-1.440	0.216
Abusers	0.977	0.120-7.942	0.983
Alcohol intake (10 g)	1.193	0.983-1.447	0.074
Hypertension (yes)	1.584	0.601-4.176	0.353
Diabetes (yes)	10.439	3.367-32.367	<0.001
Dyslipidemia (yes)	1.828	0.418-7.994	0.423
HT (yes)	2.856	0.996-8.187	0.051
LPA^5,a^			
1000-1999 cal/sem	0.867	0.159-4.737	0.870
<1000 cal/sem	0.520	0.115-2.346	0.395
BMI (kg/m^2^)	0.978	0.888-1.077	0.649
WHR ≥ 0.85^a^	3.629	1.278-10.301	0.015
WC ≥ 88 cm^a^	1.021	0.986-1.058	0.236
TC ≥ 200 mg/dL^a^	1.078	0.404-2.872	0.881
HDL-c <50 mg/dL^a^	0.472	0.152-1.464	0.194
LDL-c ≥ 160 mg/dL^a^	0.790	0.179-3.481	0.755
TG ≥ 150 mg/dL^a^	0.800	0.261-2.453	0.696
GLU ≥ 126 mg/dL^a^	3.056	0.694-13.460	0.140

^1^reference: >12 years at school. ^2^reference: premenopause. ^3^reference: nonsmoker. ^4^reference: nondrinker. ^5^reference: ≥ 2000 cal/sem. ^a^Data from 2003 interviews. P-value significance: ≤0.05. BMI: body mass index; GLU: levels of plasma glucose; HDL-c: high-density lipoprotein cholesterol; HT: hormonal therapy; LDL-c: light-density lipoprotein cholesterol; LPA: Leisure physical activity in the last year; MET: metabolic equivalent; TC: plasma total cholesterol; TG: triglycerides; WC: waist circumference; WHR: waist-to-hip ratio.

**Figure 3:**
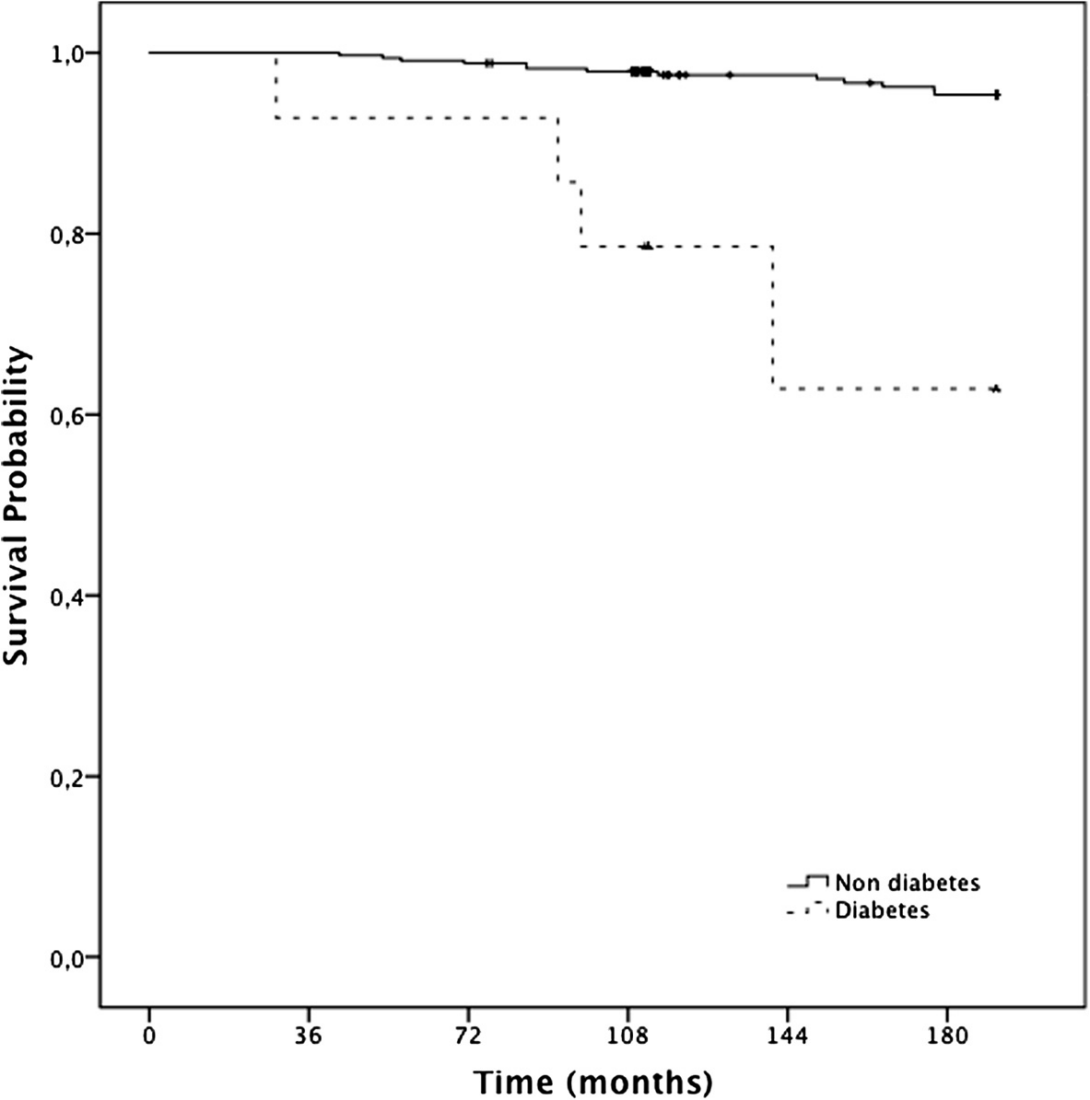
Kaplan Meier estimates of survival among women, according to diabetes prevalence (n=358).

Table 4 presents the results of multivariate analysis resulting from the Cox regression model, taking into account the confounding factors (age and smoking). Previous diagnosis of DM, WHR **≥** 0.85, and postmenopause status remained the leading risk factors for all cause mortality, similar to those found in the crude analysis and increasing the risk of death. Diabetic women had higher HR for mortality (HR = 6.645, 95% CI: 1.938-22.79, p = 0.003). Postmenopausal status tended to be associated with, and entailed a six-fold increase in, the risk of death (HR = 6.216, IC: 0.963–40.143, p = 0.055). Alcohol intake was an independent risk factor for mortality (HR = 1.228, 95% CI: 1.014-1.487, p = 0.035). The magnitude of the association between abdominal obesity (WHR ≥ 0.85) and mortality became even greater after the adjustment for BMI (HR = 2.974, 95% CI: 1.039 – 8.510, p = 0.042).Figure 3 shows Kaplan-Meier curves for DM diagnosis. The results support the findings of the adjusted model, according to which mortality is increased in the presence of diabetes.

**Table 4:** Adjusted mortality hazard ratio (HR) and 95% confidence intervals (95% CI) for survival in 358 women from a South Brazilian cohort between 1995-2010

**Predicting factor**	**HR**	**95% CI**	**p**
Diabetes	6.645	1.938-22.79	0.003
WHR ≥0.85	2.974	1.039-8.510	0.042
Menopausal status			
Perimenopause	2.260	0.656-7.784	0.196
Postmenopause	6.216	0.963-40.143	0.055
Hysterectomy	4.257	0.325-55.779	0.270
Alcohol intake (10 g)	1.228	1.014-1.487	0.035

P-value significance: ≤0.05. CI: confidence interval; HR: hazard ratio; WHR: waist-to-hip ratio.

## Discussion

To the best of our knowledge, this is the first Brazilian population-based study evaluating survival and causes of death in pre-, peri- and postmenopausal women. This prospective follow-up study including women in their middle age indicates that CVD was the main cause of mortality. In addition, postmenopausal status, DM, and central adiposity were associated with increased risk of mortality, independently of age and smoking. The present results support the findings of two previous studies investigating Brazilian postmenopausal women aged 60 to 80 years [2,3].

Coronary heart disease is the leading cause of death in women aged 60 years or older [35]. In Brazil, diseases of the circulatory system are a significant cause of mortality [19]. A study by Schmitt et al. [36] underscores that, between 1979 and 2004, cardiovascular diseases, neoplasms, and ill-defined causes were the main causes of death in women in Brazil; these results are similar to those observed in the present study. Therefore, despite the small number of deaths (4.7%), our study confirms data from previous studies, supporting the notion that CVD are the main cause of death in women in Brazil [15,17,19].

There was an increased risk of mortality in women with DM, a recognized clinical condition associated with risk for cardiovascular mortality [6,37,38]. The association between DM and CVD has been suggested to be stronger in women than in men [6,37,39]. Cardiovascular mortality is 3 to 5 times higher in diabetic compared to non-diabetic women, and 2 to 3 times higher in diabetic vs. non-diabetic men [40]. A relatively larger mortality for women with DM compared with those with prior CVD would suggest insufficient attention to CVD prevention in these women [39]. It has been suggested that physicians have a less aggressive management of CVD in women that in men, despite the greater cardiac disability in women [41]. Gender disparities are also evident both in the clinical presentation as well as misperceptions and barriers to preventive strategies [42].

It has also been suggested that diabetic women have accelerated atherogenesis. This process is not completely understood, but it is at least in part related to more severe lipid and lipoprotein abnormalities, particularly elevated levels of triglycerides and reduced levels of HDL-c, among diabetic women [43,44]. Increased levels of endothelin-1 associated with atherogenesis induce smooth muscle hypertrophy, stimulate vasoconstriction, and activate the renin-angiotensin system. Simultaneously, reduced prostacyclin and nitric oxide activity enhances platelet aggregation and adhesiveness, leading to endothelial dysfunction. These facts may contribute to the poorer outcomes in DM [45,46].

Concerning the relationship between BMI and mortality, an association between these aspects is widely accepted [12,47-49]. Even though this association was not identified in the present study, BMI is believed to be a surrogate measure of general adiposity. However, BMI measures do not distinguish between fat mass and lean mass [50]. Furthermore, changes in lifestyle patterns, such as reduction of calorie intake and increased physical activity, reduce body fat and increase muscle mass. Individuals within the overweight category may be fit and muscular rather than having excess fat [51], and a U-shaped relationship has been described, with increased mortality only at the extremes of underweight and BMI > 45 kg m [49-51]. In addition to this, data from the MacArthur Successful Aging Study suggest that WHR is the most suitable measure for risk stratification of high functioning, especially in older adults [52]. All these aspects reinforce our findings that measures of central adiposity are a good indicator of mortality rates.

The influence of HT on the risk of mortality still needs to be appropriately defined. In our study, univariate analysis showed a trend toward higher mortality among HT users, and age probably had an effect on this group. HT is considered a Class III intervention and is not effective for secondary CVD prevention in postmenopausal women [53,54]. The results from the Women’s Health Initiative study, the Heart and Estrogen/Progestin Replacement Study, and the Women’s Estrogen for Stroke Trial indicate that the use of estrogen alone or estrogen plus progestin does not prevent, and could actually increase, the risk of CVD in older postmenopausal women or in those with established CV disease [54-58]. Conversely, in women younger than 60 years and within 10 years of menopause, estrogen therapy has been associated with decreasing risk of mortality [59].

Menopausal status is an important modifier factor in female mortality [60]. Previous analyses show an association of central adiposity with postmenopausal status [22,61]. Also, when the risks of inactivity were assessed for DM, metabolic syndrome, and hypertension, stronger risk has been detected for post- compared to pre- and perimenopausal women [27]. Postmenopausal women are more prone to central adiposity [62] and development of occult DM than premenopausal individuals [60], and these risk factors are deeply linked to CVD and consequently to mortality.

Even without presenting a significant HR for death in the crude model, alcohol is a recognized risk factor linked to mortality. This was observed in the present adjusted model. A study with data from the Nurses’ Health Study demonstrates that small to moderate alcohol ingestion is related to lower mortality [10]. In a meta-analysis, a J-shaped relation was observed between mortality and alcohol intake; and this inverse association rather disappeared in women drinking lower doses than men [63]. In the present study, we found a greater risk of mortality in drinkers. This may reflect the fact that alcohol intake increases the relative risk of death in the presence of other pathologies, such as breast cancer [64].

Moreover, our study suggests that women who had more years in school had a reduction in the risk of death, as previously reported [65]. This association may be explained by the fact that women with more years in school usually seek health care, have more knowledge of prevention and thus their disease may be detected at an earlier phase with more successful outcomes. However, this difference lost significance in the multivariate analysis, perhaps because of the low number of events.

Strengths of this study are the use of a population-based cohort and a long follow-up period. Conversely, limitations include the lack of information on duration and treatment of DM and hypertension. Our analyses focus on leisure physical activity because it was the only category of physical activity measure in our 2001-2003 follow-up. This may have led to an underestimation of the level of physical activity, with misclassification (participants who were active in other types of physical activity could have a higher metabolic equivalent (MET) than that which was calculated and this would influence mortality). It is important to highlight that our sample is composed of relatively young women, which explains the low number of events (death).

## Conclusions

The present results suggest that CVD was an important cause of mortality in this cohort, and that DM and/or central adiposity were associated with all-cause mortality in a representative sample of middle-aged women from South Brazil. Lifestyle and dietary factors seem to be related to risk of mortality in middle-aged women.

## Abbreviations

BMI: Body mass index; CI: Confidence interval; CNCD: Chronic non communicable diseases; CVD: Cardiovascular disease; DM: Diabetes mellitus; GLU: Levels of plasma glucose; HR: Hazard ratio; HDL-c: High-density lipoprotein cholesterol; HT: Hormonal therapy; LDL-c: Light-density lipoprotein cholesterol; LPA: Leisure physical activity in the last year; MET: Metabolic equivalent; TC: Plasma total cholesterol; TG: Triglycerides; WHR: Waist-to-hip ratio; WC: Waist circumference.

## Competing interests

The authors declare that they have no competing interests.

## Authors’ contributions

VC, KO and PMS were involved in the conception and design of the study, data collection and analysis, and drafted the article. All the authors read and approved the final manuscript.

## Pre-publication history

The pre-publication history for this paper can be accessed here:

http://www.biomedcentral.com/1471-2458/14/194/prepub
